# Supplementary material for: Experiences of social support and the role of engagement in a digital educational support group for adolescent mothers’ health in the Dominican Republic
Source: PLOS Digit Health. 2025 Apr 21;4(4):e0000808. doi: 10.1371/journal.pdig.0000808 (PMC12011247; doi:10.1371/journal.pdig.0000808)
Supplement: Attachment — Submitted filename: Response to Reviewers.pdf [file pdig.0000808.s003.pdf]

UNIVERSITY OF CALIFORNIA SAN DIEGO

BERKELEY • DAVIS • IRVINE • LOS ANGELES • MERCED • RIVERSIDE • SAN DIEGO • SAN FRANCISCO | SANTA BARBARA • SANTA CRUZ

Herbert Wertheim School of Public Health and Human Longevity Science

9500 Gilman Drive # 0000
La Jolla, California 92093-0000
Tel: (858) 000-0000
Fax: (858) 000-0000
website.ucsd.edu

February 9, 2025

RE: Resubmission to PLOS Digital Health

Dear PLOS Digital Health Editorial Team,

Thank you for your thorough review and consideration of our manuscript. We sincerely appreciate the thoughtful feedback provided by the reviewers, which we have carefully considered while making revisions. Below we provide point-by-point responses to all reviewer comments.

Comments from reviewers:

***Reviewer #1:

- **Comment:** This is a well written manuscript presenting mixed-methods secondary analysis data. The research question is difficult to answer in a quantifiable way, this has been addressed in the discussion, and I agree a broad approach is needed.
    - **Response:** Thank you for your positive feedback on our approach and presentation of findings.

***Reviewer #2:

- **Comment:** This is an excellent manuscript on design, implementation and rigorous evaluation of an intervention for education and support of adolescent mothers in Dominican Republic. The intervention was targeted to educate and support mothers at high risk of depression, lack of future contraception and not being able to properly care for their babies due to lack of knowledge. The analysis is very detailed and rigorous. The authors realize they have a small sample size but they were able to draw valid conclusions that can help with future implementation of similar support systems. The attention to detail and rigorous both quantitative and qualitative analysis of the study are outstanding. The study demonstrates the importance of providing emotional and educational support to high risk adolescent mothers. Thank you for your submission.
    - **Response:** Thank you for your positive feedback on the content and rigor of our analysis.

***Reviewer #3:

- **Comment:** The authors present the results of an intervention to improve sexual and reproductive health in a group of adolescent mothers in Dominican Republic. I personally liked the article. It touches an important issue that disproportionally impacts those settings that have the least access to appropriate healthcare. Thus, it is an important study to inform future approaches that aim to improve health access and information across these settings.

    - **Response:** Thank you for your positive feedback on the applicability of our manuscript.
- **Comment:** The problematic described in the abstract and introduction refers to the burden of giving birth at an early age. Thus, it may seem that the goal of the study is to improve contraception in adolescent girls to reduce the rates of early motherhood. Nevertheless, all the participants are already mothers. Maybe the described problematic could further emphasize why it is important to increase the use of contraception in mothers, are the number of children significantly higher in those women that had their firstborn during adolescence? Are the markers of risk of depression and low educational attainment significantly associated with number of children? If yes, then it is important to say so. Otherwise, it is unclear why the efforts to improve contraception were focused on girls that were already mothers.
    - **Response:** We acknowledge this reviewer's concern. In response, we have revised the manuscript to provide a more logical introduction to the importance of contraception even for adolescents who are already mothers.
- **Comment:** Another confusing point regarding the usefulness of the system, and its possible impact, involves contraception accessibility. The authors say that 27% of adolescents in the included age group have to overcome numerous barriers to access contraceptives. How does their system approach this? Is it possible that the use of contraception did not improve because the mothers could not access them anyway? This is something that requires further clarification. If they cannot access contraception, then this might not be a good outcome metric for the study.
    - **Response:** We thank the reviewer for this thoughtful question. We have clarified in the methods section of this manuscript that all participants were receiving services at the MAMI clinic which provides access to contraceptives (line 270). Therefore, while access barriers are heightened among adolescents, all adolescents in this study had access to contraceptives at their healthcare facility.
- **Comment:** Regarding the association results, it is puzzling that support index and knowledge were negatively associated. Is it possible that the girls that understood best the information would interact the least because they didn't need it? The opposite could also be true. A correlation analysis between the multiple indices could be useful to better understand the results of the study.
    - **Response:** We have noted this as an additional possible explanation for these findings in our discussion (lines 634-636). We appreciate the reviewer's suggestion to add a correlation analysis between the multiple indices, but feel that adding this would lengthen the paper excessively.
- **Comment:** Finally, the authors need to improve the critical comparison of their approach to other similar approaches. Most of the end of the discussion is limited to listing other similar approaches without a quantitative comparison. It is important that the authors tell us how they are better or not than other approaches, by how much, and why. This would strengthen the impact of the paper beyond the negative results regarding contraceptive use.
    - **Response:** Unfortunately, most of the articles referenced in the discussion section of our manuscript were limited to describing patterns of user engagement and/or examining explanatory variables associated with user engagement. In our review of the literature, we found only one other article which similarly examined

how engagement was associated with a study outcome within the maternal and child health sphere. We have added a more specific report of findings from this article.

- **Comment:** Table 2 in the results is highly redundant with the corresponding text. Maybe it would be better to limit the text to describe the most important aspect of the table, while the table provides all the numerical results on its own.
    - **Response:** We have edited the corresponding text to focus more on the interpretations of each engagement metric (lines 417-425).
- **Comment:** Also regarding table 2, it does not make sense to include the immersion index as and empty row. The authors could ignore it in the table and explain in the text that the average of this metric has no meaning.
    - **Response:** We have deleted this empty row and provided this explanation in the text (lines 425-426).

***Reviewer #4:

Abstract:

- **Comment:** The objective of this section should be more explicitly stated. It is important to clearly outline the specific research question or hypothesis that this study addresses. For instance, the statement "We conducted a mixed-methods secondary data analysis" could be improved by emphasizing what the primary aim of the research was (e.g., "This study aimed to explore the mechanisms of action of the FAMA intervention through a mixed-methods secondary data analysis").
    - **Response:** We have revised this to more clearly state the objective in our manuscript abstract (lines 34-35).
- **Comment:** Provide more clarity about the methodology used. For example, the description of "linear or Poisson regression" should be expanded to explain why these methods were chosen and how they were applied in this specific context. A brief mention of how the regression models account for potential confounding factors would also enhance the clarity of the methodology.
    - **Response:** We have added more information on how statistical methods were chosen and model adjustment (lines 39-40).
- **Comment:** The results presented (e.g., "a significant positive association between engagement as measured by acknowledging intervention messages...") need to be contextualized more clearly. It would be helpful to explain what these results mean in practical terms and how they relate to the intervention's overall success. Specifically, interpreting the negative association with social support in the context of health knowledge is crucial.
    - **Response:** We have added more interpretation of our finding related to social support and health knowledge (lines 45-46).

Introduction:

- **Comment:** The introduction should include a more robust theoretical framework that underpins the study. While it does an excellent job of presenting background information, integrating relevant theories (e.g., theories of engagement, social support, or mHealth interventions) would strengthen the rationale behind the study.
    - **Response:** We thank the reviewer for this insightful comment. We have explicitly incorporated Social Support Theory as its role in this study was previously more implicit (lines 214-216). This, in addition to

our proposed mechanism of action (Figure 1), we believe provides a strong rationale underpinning our study.

- **Comment:** identification of the research gap is somewhat implicit. A more direct statement of the gap in existing literature would make the introduction stronger.
    - **Response:** We have edited language in the introduction to more explicitly identify the research gap our study addresses (lines 177-181).
- **Comment:** There are some areas where the transition between paragraphs could be smoother. Consider reorganizing the introduction to flow more logically from the global context, to the specific issues in the Dominican Republic, then to the importance of the intervention, and finally to the hypothesis.
    - **Response:** We have done significant reorganization of the introduction and incorporated smoother transitions between some paragraphs.

Materials and Methods:

- **Comment:** The description of the methods is detailed but lacks justification for some choices, such as the use of the "Engagement Index." It would be beneficial to elaborate on why this particular method was chosen to measure engagement and how it accurately reflects participant involvement in the intervention.
    - **Response:** We added more context on this, including context on WhatsApp data and its lack of metadata which might allow for a more direct measure of engagement (line 315-317).
- **Comment:** Sample description should be expanded. While eligibility criteria are provided, more context about the recruitment process and participant characteristics would help readers better understand the scope and limitations of the findings.
    - **Response:** We have added more details on the recruitment and screening process (lines 265-267).
- **Comment:** Clarify the process of data collection, particularly the integration of WhatsApp chat data with interview data. It would be useful to explain how the qualitative and quantitative data were merged, particularly the specific steps taken during the analysis to ensure that both types of data informed the interpretation of the results.
    - **Response:** We have added a more comprehensive section on the integration of qualitative and quantitative data (lines 382-390).
- **Comment:** Ethical approval and consent procedures are adequately described, but this section would benefit from further details about the confidentiality measures in place. For instance, explaining how participant anonymity was ensured when handling WhatsApp messages or interview data would strengthen the ethical transparency of the study.
    - **Response:** We have incorporated additional details on steps taken to safeguard privacy and confidentiality (lines 285-287).
- **Comment:** While statistical methods are mentioned, there is no explanation of the rationale behind choosing linear regression versus Poisson regression for different outcomes. It would be beneficial to clarify the assumptions of each model and why they were the most appropriate for the data being analyzed.
    - **Response:** We have added more explanation for our statistical analysis choices to the methods section (lines 341-345).

Results:

- **Comment:** The section lacks clarity in reporting participant characteristics. Ensure that the baseline characteristics, such as the age range, employment status, and level of education, are precisely defined. The age of participants is mentioned as a median with interquartile range, but it would be beneficial to include the full range (minimum and maximum) to provide a clearer picture of the demographic diversity.
    - **Response:** We have revised this section to include additional details on the range of participants included in our sample (lines 397-402).
- **Comment:** The engagement metrics are reported with median values and interquartile ranges but could benefit from additional statistical tests to show variability and significance. Consider using means and standard deviations where appropriate, especially when the data are approximately normally distributed. Furthermore, include p-values or confidence intervals for key comparisons to strengthen the statistical reporting.
    - **Response:** We have added mean and standard deviations to table 2, and noted which variables are not normally distributed. Table 2 displays engagement metrics descriptively. Our focus was not on comparing the values of different indices; therefore, we did not compare them statistically. Tables 5-7 include coefficients from regressions and p-values based on the association between indices and outcomes.
- **Comment:** Some message content (e.g., WhatsApp messages) is included in the Results section, but they do not appear to add value or meaningfully contribute to the data analysis. The inclusion of direct message excerpts may distract from the key findings and potentially overwhelm the reader. Consider summarizing the types of messages exchanged rather than providing lengthy quotes, unless they are directly tied to specific analysis.
    - **Response:** The purpose of including quotes from WhatsApp messages and quotes from interviews in table 4 is to validate that the functions described in the interviews were borne out in the WhatsApp groups themselves. We have added a sentence to further clarify and emphasize the consistency noted between messages and interviews on lines 525-527.

Discussion:

- **Comment:** The discussion regarding the types of social support exchanged (e.g., companionship, informational, emotional, and instrumental) could benefit from a deeper analysis. Specifically, provide a more nuanced interpretation of why companionship support predominated in the WhatsApp chats, considering the context of adolescent motherhood. Does this suggest that companionship was more vital than other forms of support due to the isolation or emotional challenges faced by young mothers?
    - **Response:** We have added a more complete discussion and interpretation of our findings related to companionship support to the discussion section (lines 589-611).
- **Comment:** The findings regarding the association between engagement and health knowledge are important but should be explored in greater depth. You mention that “greater engagement, as measured by acknowledging moderator messages with the 'emoji of the day,' was associated with higher health knowledge scores at follow-up.” Elaborate on why this form of engagement (emojis) was positively associated with health knowledge. Consider discussing potential psychological or motivational mechanisms that may explain why this form of engagement, which appears less substantive, was more meaningful for the participants than more consistent messaging.

- **Response:** We discuss this on lines 622-629:
  - *"This may reflect that sending the accurate emoji of the day involves a closer reading of the daily informational message, whereas sending a message each day could have been achieved by sending a greeting in the morning without engaging with any of the informational material from the intervention."*

- **Comment:** The finding of a negative association between the Support Index and health knowledge warrants further exploration. Provide a detailed discussion of possible explanations for this unexpected result. Could it be that participants who engaged more in social support exchanges were less focused on the informational content? Was there a distraction effect caused by the peer-to-peer interaction, and if so, how might future interventions better balance companionship with educational content?
  - **Response:** We have added discussion around the potential for participants to have been distracted by social support exchanges, and how future interventions may approach this issue in the future (lines 633-634, 752-756).
- **Comment:** The study identifies "adherence" as a form of engagement, yet it's not entirely clear why this particular measure was significant in predicting health knowledge scores. The discussion should clarify why this specific type of engagement (adhering to the moderator's daily prompts) was associated with improved health knowledge, and how this can inform future interventions. Do participants who show high adherence to prompts tend to engage more meaningfully with the content, or is this association merely a byproduct of the structure of the intervention?
  - **Response:** We discuss a possible explanation for why this type of engagement may be associated with health knowledge on lines 622-629, and how future interventionists may leverage these results on lines 697-699.
- **Comment:** The study's results contrast with previous findings, where FAMA was associated with increased health knowledge and contraceptive use. In the discussion, acknowledge this discrepancy and analyze potential reasons for this divergence. Were there external factors at play that could have impacted the outcomes (e.g., cultural differences, sample characteristics)? Provide a more thorough comparison with existing literature to situate your findings within the broader context of digital health interventions.
  - **Response:** The findings of this study do not contrast with the original study findings: the original study evaluated pre-post changes in outcomes, whereas our focus was on how engagement was associated with study outcomes. We have added some text throughout our manuscript to clarify the differences. We have also incorporated additional literature and concrete findings from a similar study to our discussion section (lines 673-676).
- **Comment:** The limitations section is somewhat brief and should be expanded. For example, the small sample size and the single-clinic design are mentioned, but how might these limitations specifically impact the generalizability of the results? Were there any biases in participant selection or in the completion of outcome measures (e.g., who dropped out or who missed surveys)? Discuss how these limitations could affect the interpretation of the findings and suggest avenues for future research to address these gaps.

- **Response:** We have significantly expanded the paragraph on study limitations and their impact on our interpretations of findings (lines 707-744).

- **Comment:** The discussion could be strengthened by including more specific recommendations for future studies. Based on the results, what additional data or approaches would be valuable to explore? Consider suggesting longitudinal studies to better assess the long-term impact of digital interventions on maternal health outcomes, or exploring alternative ways of measuring engagement that go beyond text-based communication (e.g., multimedia interactions, passive reading of content).
  - **Response:** We have added more suggestions for future research in this area (lines 761-766).

***Reviewer #5:

- **Comment:** I would like to say first and foremost I enjoyed reading your article and the type of research you are doing. This research is so crucial for adolescent mothers and with the increased use of digital health to support knowledge acquisition and increased connections/communications with patients, this understanding of what needs they need help with is timely and pertinent. To this I agree findings from your manuscript would assist other clinics globally with helping this target population (generally but there would naturally be cultural variations).
  - **Response:** Thank you for this positive review of our research, and recognition of its utility.
- **Comment:** One of the struggles I believe you experienced was low power due to the low N of subjects enrolled and then the reduced numbers of outcome data received. This most likely impacted the effect size with finding the strength of relationships between variables or how large that difference was. As the power appears low, which is suggested by the low levels of statistical significance, I would be very careful to state your findings are conclusive but rather important findings that warrant further attention and research analysis. This is particularly true for Lines 425-427 "Our finding of significant association between a measure of engagement and health knowledge score bolsters the prior findings by supporting a dose-response relationship between intervention engagement and increased knowledge". Because of the low N, it gets very tricky to state a conclusive exposure-response relationship as this would require a larger N or power from the study.
  - **Response:** We have adjusted our language accordingly and added more language around the limitations of our sample size in the discussion section (lines 613-618).
- **Comment:** Knowledge acquisition may improve skills but changing behaviors is more complicated. Thus, to change a behavior, finding individual's core needs and motivations for change will support increased engagement and ultimately attitude change.
  - **Response:** We fully agree with this statement and believe this aligns well with our hypothesized mechanism of action.
- **Comment:** Line 83- "Social support is key for adolescent mothers whose developmental stage is characterized by increased reliance on and trust of peers" Can you explain by the publications referenced how social support is key? Can you back it up possibly with a published quantitative value? Lines 84-86- "Adequate social support, particularly peer support has been associated with reduced perinatal depression, improved knowledge about child development, improved breastfeeding indicators" Can you provide the published numbers of reduction or improvement?

- **Response:** We have added references to observed quantitative changes in maternal health outcomes associated with peer social support in the introduction section (lines 161-163).

- **Comment:** From Lines 382-383- Findings Support Index and the Adherence Index were individually associated with health knowledge scores. This warrants further understanding from your population – what were the factors within the social support that influenced their adherence or engagement and eventually supported knowledge acquisition. What changed their attitudes? Was anything found from the interviews about this? If not, can you go back and ask?
    - **Response:** Thank you for this thoughtful comment. Unfortunately, participants did not comment on this in interviews, and the study has since closed so we are unable to contact the participants.
- **Comment:** Lines 393-394 "We found no significant association between the Engagement Index and post-intervention social support scores". I think the types of engagement got a bit mixed together since the focus seemed more on knowledge acquisition.
    - **Response:** Thank you for this comment. We did however also test whether engagement with the intervention was associated with social support scores post intervention.
- **Comment:** Lines 399-400 – "In qualitative interviews, participants highlighted the importance of informational and emotional support more often than was reflective of the observed frequency of these messages" This warrants further investigation and an important point as this could suggest what you are designing maybe missing their actual needs. A more detailed needs assessment could be conducted from this preliminary study. It shows that how you perceive success with the app may be different from how the target population perceives it as helpful and what they actually need. For example, in the Author summary you actually mentioned this-"future digital group interventions should consider diverse engagement styles and the specific needs of adolescent mothers." I was hoping to get a better understanding of this in the manuscript.
    - **Response:** On lines 695-698 we have further expanded on this finding of small discrepancies between sources of data, and how it may be helpful for future research.
- **Comment:** Lines 419-421 "We found that greater intervention engagement, as measured by acknowledging moderator messages with the "emoji of the day" (adherence) was associated with higher health knowledge scores at follow-up." I think it would hard to conclude if A then B. The higher health knowledge scores were noted but could you expand more on how you tested their knowledge to truly know if knowledge was attained? Some could have just "skimmed" they article to get to the emoji and then send the emoji in the message. Also, as you mentioned you do not know if they read the articles and then forgot to send the emoji. Did you ever ask them? Were you able to get a rough number per participant of read but non-emoji responses? This is the same point here in Lines 458-459, "Our analysis extends these findings to suggest that a close reading of message content has a greater effect on knowledge than other forms of engagement."
    - **Response:** We thank you for this comment and wish to clarify here that we are discussing knowledge based on pre and posttests of health knowledge related to intervention content (see lines 291-294). We have also expanded on the limitations we faced in terms of potentially misclassifying participant adherence engagement in both directions to the discussion section (lines 728-736).

- **Comment:** Lines 466-467- “This study incorporated engagement metrics such as frequency of app use, number of library articles read in the app, and user feedback on satisfaction”. This could be explained in the methods section and design of the app in the introduction to help explain the knowledge the participants received.
    - **Response:** We believe there was some confusion as these lines referred to one of the studies cited in the discussion section. We have added some additional language to further clarify this (line 671).
- **Comment:** You have tapped into something here with helping this target population who desire a community or a place where they feel they belong and are accepted especially since they are at risk for subjugation to stigmas and discrimination. Correlating to feeling accepted or belonging to a group (adolescent behavior of love and connection- need for social connection), can you reference further any data you may have collected on depression/postpartum depression, social isolation, and loneliness? Can you expand on these points? Did you collect any data on increased mother-baby bonding?
    - **Response:** Although the need and appreciation for social connectedness was salient in post intervention interviews, this study did not gather data on depression, post-partum depression, or mother-baby bonding. However, social isolation is one of five scales combined in our measure of perceived social support. In the original study findings, we found that perceived social isolation did not significantly change from pre to post test.
- **Comment:** Line 408-409 –“Overcoming the isolation of adolescent motherhood by connecting with peers was a vital part of the appeal of this intervention to participants”- this needs to be expanded further in the article to provide the backup for its significance.
    - **Response:** We have added a sentence in the results section (lines 496-498) and clarified in the discussion section that this is based on what participants reported in interviews (line 593).
- **Comment:** Lines 495-496 “some members decided to make another, separate chat group just for participant messaging, with no rules and without the participation of the moderators”. This I can imagine was frustrating as this was lost data. Going forward, it would be helpful to state in the informed consent that groups can be developed after the completion of the intervention but not during as the point is to help them. But their feedback is welcome at any point- could be done as an anonymous message or survey form for quality improvement.
    - **Response:** We thank you for this thoughtful suggestion. This comment will be shared back with the MAMI staff.
- **Comment:** Lines 507-508 “Interventionists should also consider the need for companionship among young mothers yet recognize its potentially neutral (or even negative) role in relation to changes in health knowledge.” This needs to be expanded further so explain how this conclusion was drawn.
    - **Response:** We have added a significant amount of discussion around this to our discussion section to clarify why this conclusion was drawn (lines 589-611).
- **Comment:** This really is an important point and how our social cognition is changing to two different versions- one from having “in person” contact and the one from digital contact and online social exchanges. How will you be able to detect if a mother is experiencing post-partum depression and then possibly has negative interactions with a participant online. How would you be able to detect an issue or protect them from this? There is a great article I

would recommend reading - Social cognitive neuroscience in the digital age - PubMed (DOI: 10.3389/fnhum.2023.1168788)

- **Response:** Thank you for this thoughtful comment on the potential risks inherent in digital interventions. We have added reference to the potential risks on lines 762-763.

We believe these revisions effectively address all reviewer concerns, and we hope the revised manuscript meets the standards for publication. Again, we sincerely appreciate the reviewers' time and thoughtful feedback, which have significantly improved the manuscript.

Thank you for considering our manuscript for publication.

Sincerely,

Alana Lopez, MPH
Joint Doctoral Program – Health Behavior
University of California San Diego
